# Osteoarthritis and functional disability: results of a cross sectional study among primary care patients in Germany

**DOI:** 10.1186/1471-2474-8-79

**Published:** 2007-08-08

**Authors:** Thomas Rosemann, Gunter Laux, Thomas Kuehlein

**Affiliations:** 1 University Hospital Heidelberg, Department of General Practice and Health Services Research, Voßstr. 2, 69115 Heidelberg, Germany

## Abstract

**Background:**

The aim of the study was to determine factors associated with functional disability in patients with OA.

**Methods:**

1250 questionnaires were distributed to OA outpatients from 75 general practices; 1021 (81.6%) were returned. Questionnaires included sociodemographic data, the short form of the Arthritis Impact Measurement Scale (AIMS2-SF), and the Patient Health Questionnaire (PHQ-9) to assess concomitant depression. A hierarchical stepwise multiple regression analysis with the AIMS2-SF dimension "lower body" as dependent was performed.

**Results:**

Main factors associated with functional disability were depression symptoms, as reflected in a high score of the PHQ-9 (beta = 0.446; p < 0.0009), pain as reflected in the AIMS2-SF symptom scale (beta = 0.412; p = 0.001), and few social contacts (beta = 0.201; p < 0.042). A high body mass index was associated with lower functional ability (beta = 0.332; p = 0.005) whereas a higher educational level (beta = -0.279; p = 0.029) predicted less impairment. Increased age was a weak predictor (beta = 0.178; p = 0.001) of disability. With a p of 0.062 the radiological severity according to the grading of Kellgren and Lawrence slightly surpassed the required significance level for remaining in the final regression model.

**Conclusion:**

The results emphasize that psychological as well as physical factors need to be addressed similarly to improve functional ability of patients suffering from OA. More research with multifaceted and tailored interventions is needed to determine how these factors can be targeted appropriately.

## Background

Disability has a substantial impact on the quality of life of patients and affects daily living [1]. It is associated with extensive direct and indirect costs and represents a considerable burden for the health care system and the society in general. In the US, direct medical costs for persons with disability were estimated to be $260 billion during 1996 [2]. The leading reason for disability in the general population is osteoarthritis (OA) [3,4]. Data from the Centers for Disease Control (CDC) show that approximately 1 in 3 adults (37.6%) with arthritis reported limitation in their usual activities [5]. In the general population data from the Nottingham study, 14% of subjects in the age group of 40–79 years had knee pain with disability on most days of the previous month [6]. Due to the aging of most societies in the western world, the high prevalence of OA is expected to increase further in the upcoming years [7]. Interestingly, the degree of disability varies widely in subjects with the same degree of pathomorphological damage to the joint [8-10]. Prior studies focused on the question to what extent arthritis contributes to the risk of developing difficulties in activities of daily living (ADL) and revealed multiple factors: Song and colleagues for instance found that arthritis is associated with an odds ratio of 1.5 (95% CI 1.2–1.8) to develop ADL disability [1]. Obviously, many factors influence functional disability of patients with OA. Comorbidities as for example depression, which is highly prevalent among OA patients [11], can be assumed to contribute substantially [12-14]. Even though the causality of the association is yet unclear, the relation seems to be bidirectional: depression leads to decreased physical activity and functional limitation is associated with increased prevalence of depression. Since it is obvious that many factors contribute to functional disability, studies assessing functional disability need to control a variety of factors. Therefore, in this study, in a large sample of OA patients in primary care, factors associated with functional disability shall be assessed which have not yet been assessed altogether, i.e. depression and other comorbidities, obesity, duration of OA and amount of physical activity.

## Methods

The data analyzed in this study were part of the baseline assessment of the PraxArt project. The project is financed by the German Ministry for Education and Research over a period of six years starting from 2003 and comprises data from a representative sample of 75 general practitioners' (GPs) practices in the area of Baden-Wuerttemberg and Bavaria. The project aims at learning about needs of patients with OA and at developing tailored multifaceted interventions to increase quality of life [15]. The Ethical Board of the University of Heidelberg approved the study. The study protocol has been published elsewhere [15].

### Patient inclusion criteria

To be eligible for inclusion, patients had to be adult (18 years or older) and diagnosed with OA of the hip or the knee according to the ACR criteria [16]. A further criterion for inclusion was OA as the reason for the current encounter in order to avoid overrepresentation of patients with other chronic diseases and OA as comorbidity. According to that criterion, in each of the 75 practices 15 patients were contacted in consecutive order of appearance and informed about the option to participate in the study.

### Data collection

After giving their written informed consent, patients received a package of questionnaires and a stamped envelope with the postal address of the university. They were asked to return the questionnaires to the university and were informed that neither the GP nor the practice team had any possibility to get knowledge of the patients' answers. All patients received written reminders, but were also reminded by their GP to return the questionnaires.

Each questionnaire was linked to the participants list with an identification number, so data given by patients could be checked by comparing them with the patients' medical files. This procedure was done for all patients regarding all data available at the medical file, since it was the aim to assess accuracy of self reports later on in the project. If differences occurred between the data sources, the data from the medical file were used for calculations. Since GPs were asked to also note patients who declined participating, it was possible to compare the non-responders to the participants with regard to most sociodemographic variables, comorbidities and medication.

Collected sociodemographic data included sex, age, ethnicity, educational level (defined as follows: 1≤ elementary school; 2 = high school; 3 ≥ college degree), working situation (1 = unemployed or retired, 2 = half time, 3 = fulltime) and marital status (1 = living alone, 2 = married/living with partner).

#### Comorbidities

Since comorbidities may be of substantial influence on functional ability [13], the following ten comorbid conditions were controlled: high blood pressure (defined as >140/90 mmHg), diabetes type I and II, heart insufficiency (HI), coronary vessel disease (CVD), elevated cholesterol level (defined as total Cholesterol > 200 mg/dl), ulcer or stomach disease, asthma/chronic obstructive pulmonary disease (COPD), renal insufficiency, (history of) cancer and stroke.

#### Depressive disorders

Depression has a detrimental effect on functional ability [17] and shows increased prevalence among OA patients [11]. Depression symptoms were assessed by means of the depression module of the German form of the Patient Health Questionnaire (PHQ-9) [18,19]. The PHQ-9 is a completely self-administered questionnaire that enables screening for depression and assessment of depression severity [20]. For each of the 9 depressive symptoms, which accord to the Diagnostic and Statistical Manual of Mental Disorders, 4^th ^edition (DSM-IV) [21], patients indicated whether, during the previous 2 weeks, the symptom had bothered them "not at all," "several days," "more than half the days," or "nearly every day," yielding a score of 0 to 3. The PHQ-9 severity score thus ranges from 0 to 27. The internal reliability of the PHQ-9 severity measure is excellent with a Cronbach's α between 0.86 and 0.89 [22,23]. The PHQ-9 has proven to be a valid instrument for those assessments [24,25]. Patients achieving a score equal or above 15 are regarded as suffering from at least minor depression.

#### Quality of Life

The most widespread instrument to assess quality of live (QoL) in OA patients is the Arthritis Impact Measurement Scale (AIMS), available also in a revised short form, the AIMS2-SF [26-28]. It represents a reliable, valid, and comprehensive tool and has recently been validated in German in a sample of OA patients [29]. It covers the five dimensions physical limitation (separated in upper body and lower body), affect, social, work and symptom. The symptom scale reflects pain intensity [30] and has frequently been used to assess pain in intervention trials [31,32]. The social scale is defined as reflecting patients' social interactions and network. It contains 4 items asking about the frequency of personal contacts with friends and relatives (including phone contacts) but also if relatives and friends are sensitive to the individuals' needs. The affect scale reflects patients' mood, without assessing depression as an outstanding disease. The work scale assesses restrictions in daily work caused by OA and is therefore applicable only to patients who are not retired. Scale internal validity and reliability of all scales of the AIMS2-SF are excellent [29]. Possible scores range from 0–10, with 10 indicating worst health.

#### Physical activity

Physical activity (PA) has a positive influence on the course of OA [33,34], therefore we assessed PA by means of the International Physical Activity Questionnaire (IPAQ). The IPAQ was developed by an international panel of experts (EUPASS), validated in nine European countries, including Germany, and has frequently been used to assess physical activity in different countries [35,36]. Its activity categories are adjusted to the recommendations of the Centers for Disease Control [37]. The physical activity related energy expenditures (MET-min/week) were calculated according to the recommendations of the IPAQ Executive Committee for the 7-days short-version. For vigorous physical activity, the total minutes per week were multiplied with the factor 8, for moderate physical activity with the factor 4, and for walking with the factor 3.3. The sum of these three products is the MET-min/week.

### Radiographic severity

Severity of radiographic changes were classified according to Kellgren et al. [38] by a radiologist. Only x-rays not older than six months at the time of inclusion were scored and included in the analysis.

### Data analysis

The data were analyzed with SPSS (version 12.0). Descriptive analyses included continuous variables, reported with means and standard deviations (SD). T-tests were used for group comparisons. For dichotomous/categorial variables, absolute numbers and percentages were displayed and comparisons were made by means of Fishers' exact test. Bivariate correlations of the respective variable with the lower body scale (which represented the dependent variable in the regression analysis later on) were calculated. Since a linear relationship could not be assumed for at least some of the variables, this was done by means of Spearman's rho. Factors entered into the regression model were chosen according to the achieved significance in the correlation analysis (p <= 0.05). To assess predictors, different types of multiple linear regression models are available. We chose a hierarchical stepwise technique with sociodemographic variables entered in the first block with subsequent backward elimination. This was done to avoid an artificially high *R*^2 ^due to forced entry of highly correlated factors into the model. Multicollinearity could be assumed at least for some of the factors, so that other approaches would have resulted in a large number of factors [39-41].

## Results

Overall, the GPs addressed 1311 patients, 1250 (95.3%) agreed to receive the package of questionnaires. The main reason given for not participating was time effort. Finally, 1021 of the distributed 1250 questionnaires (81.7%) were returned and analyzed. As far as data were available in patient files, the non-respondents were compared to the respondents. In terms of sociodemographic variables as age and gender, as well as in available disease characteristics (as duration of disease, number of comorbidities, health service utilization, prescription of pain relievers), both groups did not differ significantly. Over 90% of the enrolled patients were Caucasians. Missing data occurred mainly within the same package of questionnaires, in total in 271 of the 1021 questionnaires. In 123 cases the data could be completed by means of the patients' medical files. Most missing data occurred in the IPAQ (148, 14.5% of questionnaires), whereas the PHQ-9 was completed by 344 men (98.9%) and 668 women (99.1%). For 448 women (66.5%) and 287 men (82.7%) a radiological scoring according to Kellgren was available.

Characteristics of the study sample, separated by gender, are displayed in table 1. Gender distribution in the study sample was in accordance with the prevalence of OA in the general population: 674 (66.0%) of the enrolled patients were female. Most of them were already retired from work (67.1% of men and 71.5% of women). Men lived significantly more often in a partnership than women did. Regarding the BMI, age, number of comorbidities and duration of disease, no significant differences between men and women occurred. Interestingly however, QoL was more limited among women, reflected in significantly higher scores in the symptom scale, the lower body scale and the affect scale.

**Table 1:** Characteristics of the study sample separated by gender (n = 1021)

	Gender			
	
	Male (347/34.0%)		Female (674/66.0%)	
	
	mean	SD/%	mean	SD/%
Age	65.16	14.75	66.64	15.33
Duration of OA (years)	14.80	16.18	13.13	11.09
Body Mass Index (BMI)	28.39	4.26	28.12	5.16
Educational Level (1–3)**	2.61	1.11	2.38	0.83
Married/Living in partnership**	278	80.1 (%)	376	55.8 (%)
Retired	233	67.1 (%)	482	71.5 (%)
Radiographic severity (Kellgren score)*	2.76	0.92	2.53	0.77
IPAQ score (MET/week)	2356.2	(1982.5)	2108.3	(1879.6)
No. of comorbid conditions (0–10)	2.20	1.81	2.24	1.65
PHQ-9 score	15.33	4.76	15.95	4.63
Comorbidities	n	%	n	%
High blood pressure**	181	52.1	384	56.9
Elevated cholesterol	124	35.7	245	36.3
Diabetes	57	16.4	120	17.8
Chronic Heart Failure	63	18.1	131	19.4
CVD	62	17.8	70	10.3
Ulcer/Gastritis	77	22.1	146	21.6
Asthma/COPD	34	9.8	64	9.5
Renal Insufficiency	23	6.6	33	4.9
Cancer	21	6.1	16	2.4
Stroke	16	4.6	30	4.4
Quality of life (AIMS2-SF dimensions):				
Lower body **	2.39	1.71	2.98	2.08
Upper body	1.38	2.33	1.54	2.22
Symptom **	4.49	2.17	5.12	2.18
Affect **	2.60	1.28	3.10	1.36
Social	4.75	1.95	4.61	1.76
Work * (126 women/89 men)	3.08	2.67	2.34	2.23

p < 0.05; ** p < 0.01 in group comparison (t-test, Fishers exact test respectively); Comparison of AIMS2-SF dimension by means of ANCOVA, adjusted for age, duration of disease, no. of comorbidities and PHQ-9 score

The localization of OA, based on clinical diagnosis and x-rays where available is displayed in table 2. As can be seen, of the 347 men, 191 (55.0%) suffered from OA of the hip, whereas among women only 236 (35.0%) had hip OA.

**Table 2:** Localization of OA within the study sample

	total	Hip		Knee	
		Men	Women	Men	Women

Number of participants	1021	191	236	156	438
Unilateral OA (%)	150 (14.7)	32 (16.7)	41 (17.3)	32 (20.5)	45 (10.2)
Bilateral OA (%)	871 (85.3)	159 (83.2)	195 (82.6)	124 (79.5)	393 (89.7)

To assess the significance of correlations, Spearmans' rho was calculated for bivariate correlations between sociodemographic variables, disease characteristics and other data with the lower body scale. Results are displayed in table 3. Significant correlations (p < 0.01) occurred in all sociodemographic variables, i.e. gender, age, educational level, and marital status. However, Spearman's rho for all sociodemographic characteristics was quite low (0.136–0.142), indicating only weak correlations. The correlation with other characteristics were higher and ranged from rho = 0.159 for the BMI to rho = 0.515 for the symptom scale. Interestingly, the correlation with the number of comorbidities (rho = 0.177) was lower than the correlation with the PHQ-9 score (rho = 0.445). With a rho of 0.298, the correlation with the radiological grading was notable low.

**Table 3:** Correlations of patients' demographic and clinical variables with the lower body scale of AIMS2-SF

		Lower body scale Spearman rho
Gender**		0.121
Age*		0.098
Education**		-0.137
Marital status*		-0.081
Disease duration**		0.177
BMI**		0.159
IPAQ score (MET/week)**		0.136
Kellgren score*		0.298
No. of comorbidities**		0.138
PHQ-9 sum score**		0.445
AIMS 2-SF scales	Upper body*	0.210
	Symptom**	0.515
	Affect**	0.470
	Social**	0.199

Level of significance: *p < 0.05 **p < 0.01;

Table 4 displays the results of the hierarchical stepwise regression with the lower body scale of the AIMS2-SF as the dependent variable. The adjusted R^2 ^of 0.502 indicates that the remaining factors are able to explain half of the variance in the dependent variable. The PHQ-9 score (beta = 0.446; p = 0.001) remained in the model as the strongest predictor for decreased functional ability. Patients' perceived pain was the second strongest predictor (beta = 0.412; p = 0.001). A higher educational level was associated with lower scores in the lower limb scale (-0.279; p = 0.029) whereas a higher BMI was related to a higher score in the lower body scale (beta = 0.332; p = 0.005) and a higher social scale value, indicating less social contacts (beta = 0.201; p = 0.042). The contribution of age to the functional limitation at the lower limb was comparatively small with a beta of 0.178 (p = 0.001). Interestingly, the Kellgren score was eliminated in the last step of the regression, slightly surpassing the required significance level of 0.05 with a p of 0.062 and a beta of 0.155.

**Table 4:** Predictors of functional impairment of the lower body assessed by stepwise multiple regression model

Dependent: Symptom Unadjusted R^2 ^= 0.502 Adjusted R^2 ^= 0.478 F = 18.02; p < 0.0001	B	SE	T	P
PHQ-9 score	0.446	0.117	3.960	<0.0009
Symptom*	0.412	0.114	3.064	0.001
Educational level	-0.279	0.149	-2.092	0.029
BMI	0.332	0.072	2.022	0.005
Social*	0.201	0.098	2.034	0.042
Age	0.178	0.102	1.998	0.001

* AIMS2-SF scale

## Discussion

Depression, pain intensity, educational level, BMI and social contacts are the most important factors associated with disability of the lower limb in patients suffering from osteoarthritis of the knee or hip.

The impact of depression on physical activity is well known. A study among patients with rheumatoid arthritis even revealed a linear relationship between depression severity and functional disability [42]. Even though the causality of the relationship remains unclear, it seems to be bidirectional [17,43]. Besides its relation to disability, depression also has a substantial effect on the perception of pain [44].

Since significant more men than women in our study sample were married or lived in a partnership, which could be correlated to the prevalence of depression, we assessed a possible correlation between the PHQ-9 score and the marital status. Spearman's rho for the correlation between "being married/living in a partnership" and the PHQ-9 score was -0.046, but this correlation achieved no significance (p = 0.191). Consequently, the differences in the marital status between gender in our study sample can be excluded as confounder for our results.

Our study again emphasizes the need to stop the vicious circle of depression, pain and functional disability in order to increase physical activity and muscle strength. Physical activity and muscle strength are known to have a positive influence on the course of OA [45]. Interestingly, even if the regression coefficient beta was similar, depression was a stronger predictor for disability than pain. One reason might be that pain in OA is mostly not constant but shows peaks e.g. after intense activity.

The finding that depressed mood and/or depression increases pain perception in patients suffering from musculoskeletal disorders has been known for a long time [46,47]. Since several studies have pointed out the importance of the social network for coping with chronic diseases and especially with pain, our results are plausible [42,48,49]. Even simple interventions, providing some kind of social support, as for instance monthly telephone calls, have shown to improve patients' QoL [50]. And interventions for osteoarthritis patients, involving for instance the spouse, have shown to achieve better results [51].

Increased body weight is one of the major risk factors for developing OA, but also for aggravation of joint damage and related symptoms [52]. Therefore it seems quite plausible that the BMI is associated with functional impairment [52,53]. Radiological severity of OA has shown to be correlated with the BMI, especially in women [54]. Nevertheless, the fact that the Kellgren score did not remain in the final model by slightly surpassing the required significance displays that the impact of the BMI on disability in our study sample was more significant. The fact that the educational level remained in the regression model as a predictor is in accordance with prior findings emphasizing the importance of a higher education level for coping strategies and managing daily live in chronic diseases [55]. Interestingly, in OA a low educational level has been found to be associated with both more severe radiographic findings as well as more pain in the knee [56]. However, it has to be considered that these effects could be due to barriers in accessing the health care system [44].

An interesting finding is the fact that the social network contributes to physical activity. This result is supported by previous findings showing that interventions offered in a group setting or involving the spouse result in increased success of these programs [51]. A possible correlation between the marital status and social contacts would represent a possible confounder for our findings, consequently we assessed this correlation but no significance could be revealed.

Prevalence of OA increases with age [57]. Moreover, since OA is a degenerative disease, severity of pain and functional disability increase. This was confirmed by our findings and is in line with prior research where age was also revealed as predictor for increased disability in OA [1].

Some weaknesses of the study have to be noted. Due to the cross-sectional design, the revealed factors could not be confirmed as predictors over time. The fact that a control group with people without OA was not available may have led to an underestimation of the impact of OA. Furthermore, we did not assess factors as for instance self-efficacy, which showed to contribute to the course of the disease and functional ability [46]. Because of the large number of different questionnaires already used in this study, we abandoned including further instruments to assess self-efficacy. The study population had a remarkably high educational level, a finding which is mainly due to the setting in private solo practices. This may limit the comparison with data collected in hospital settings or Health Maintenance Organizations (HMO). Even though many findings of this study confirm prior findings, it is superior to prior studies in some terms: No other study before assessed    more factors including depression simultaneously. It may therefore give a more realistic reflection of the situation in primary care.

In conclusion, our results confirm in particular prior findings as those by Sharma et al. [33] who found baseline laxity, knee pain intensity, self-efficacy and social support as predictors for decreased functional ability. Contrary to their findings, increased physical activity was not associated with a decreased risk of disability in our study sample. An explanation might be that different assessment instruments for physical activity were used. A new aspect revealed in this study was the substantial impact of depression on disability among OA patients. None of the prior studies among OA patients did control for depression. Furthermore, in an average sample of primary care patients our data showed the small predictive value of x-rays for individuals' complaints and for disability and confirmed once again the weak association between structural changes and functional disability.

## Conclusion

As in rheumatoid arthritis [58], our results emphasize that psychological as well as physical factors need to be addressed similarly in aiming at improving functional ability. More research with multifaceted and tailored interventions is needed to determine how these factors can be targeted appropriately.

## Competing interests

The author(s) declare that they have no competing interests.

## Authors' contributions

TR conceived and performed the study and drafted the manuscript. GL performed the data management and statistical calculations. TK participated in conceiving the study design and in revising the manuscript. All authors read and approved the final manuscript.

## Pre-publication history

The pre-publication history for this paper can be accessed here:



## References

[B1] Song J, Chang RW, Dunlop DD (2006). Population impact of arthritis on disability in older adults. Arthritis Rheum.

[B2] (2001). Prevalence of disabilities and associated health conditions among adults--United States, 1999. MMWR Morb Mortal Wkly Rep.

[B3] Dominick KL, Ahern FM, Gold CH, Heller DA (2004). Health-related quality of life and health service use among older adults with osteoarthritis. Arthritis Rheum.

[B4] Peat G, McCarney R, Croft P (2001). Knee pain and osteoarthritis in older adults: a review of community burden and current use of primary health care. Ann Rheum Dis.

[B5] (2005). Racial/ethnic differences in the prevalence and impact of doctor-diagnosed arthritis--United States, 2002. MMWR Morb Mortal Wkly Rep.

[B6] Parker CJ, Morgan K, Dewey ME (1997). Physical illness and disability among elderly people in England and Wales: the Medical Research Council Cognitive Function and Ageing Study. The Analysis Group. J Epidemiol Community Health.

[B7] Woolf AD, Pfleger B (2003). Burden of major musculoskeletal conditions. Bull World Health Organ.

[B8] HA V, Peyron  (1980). Clinical versus radiological osteoarthritis in the general population. Epidemiology of osteoarthritis.

[B9] Dieppe PA (2004). Relationship between symptoms and structural change in osteoarthritis. what are the important targets for osteoarthritis therapy?. J Rheumatol Suppl.

[B10] Hakala M, Nieminen P, Manelius J (1994). Joint impairment is strongly correlated with disability measured by self-report questionnaires. Functional status assessment of individuals with rheumatoid arthritis in a population based series. J Rheumatol.

[B11] Rosemann T, Backenstrass M, Joest K, Rosemann A, Szecsenyi J, Laux G (2007). Predictors of depression in a sample of 1,021 primary care patients with osteoarthritis. Arthritis Rheum.

[B12] Arnow BA, Hunkeler EM, Blasey CM, Lee J, Constantino MJ, Fireman B, Kraemer HC, Dea R, Robinson R, Hayward C (2006). Comorbid depression, chronic pain, and disability in primary care. Psychosom Med.

[B13] Dunlop DD, Lyons JS, Manheim LM, Song J, Chang RW (2004). Arthritis and heart disease as risk factors for major depression: the role of functional limitation. Med Care.

[B14] Krishnan E, Hakkinen A, Sokka T, Hannonen P (2005). Impact of age and comorbidities on the criteria for remission and response in rheumatoid arthritis. Ann Rheum Dis.

[B15] Rosemann T, Korner T, Wensing M, Gensichen J, Muth C, Joos S, Szecsenyi J (2005). Rationale, design and conduct of a comprehensive evaluation of a primary care based intervention to improve the quality of life of osteoarthritis patients. The PraxArt-project: a cluster randomized controlled trial [ISRCTN87252339]. BMC Public Health.

[B16] Altman R, Alarcon G, Appelrouth D, Bloch D, Borenstein D, Brandt K, Brown C, Cooke TD, Daniel W, Feldman D, . (1991). The American College of Rheumatology criteria for the classification and reporting of osteoarthritis of the hip. Arthritis Rheum.

[B17] Alexopoulos GS (2005). Depression in the elderly. Lancet.

[B18] Spitzer RL, Kroenke K, Williams JB (1999). Validation and utility of a self-report version of PRIME-MD: the PHQ primary care study. Primary Care Evaluation of Mental Disorders. Patient Health Questionnaire. JAMA.

[B19] Lowe B, Grafe K, Zipfel S, Witte S, Loerch B, Herzog W (2004). Diagnosing ICD-10 depressive episodes: superior criterion validity of the Patient Health Questionnaire. Psychother Psychosom.

[B20] Nease DE, Maloin JM (2003). Depression screening: a practical strategy. J Fam Pract.

[B21] Association AP, Association AP (1994). Diagnostic and statistical manual of mental  disorders Ed 4.

[B22] Kroenke K, Spitzer RL, Williams JB (2001). The PHQ-9: validity of a brief depression severity measure. J Gen Intern Med.

[B23] Lowe B, Spitzer RL, Grafe K, Kroenke K, Quenter A, Zipfel S, Buchholz C, Witte S, Herzog W (2004). Comparative validity of three screening questionnaires for DSM-IV depressive disorders and physicians' diagnoses. J Affect Disord.

[B24] Lowe B, Kroenke K, Herzog W, Grafe K (2004). Measuring depression outcome with a brief self-report instrument: sensitivity to change of the Patient Health Questionnaire (PHQ-9). J Affect Disord.

[B25] Backenstrass M, Frank A, Joest K, Hingmann S, Mundt C, Kronmuller KT (2006). A comparative study of nonspecific depressive symptoms and minor depression regarding functional impairment and associated characteristics in primary care. Compr Psychiatry.

[B26] Meenan RF, Mason JH, Anderson JJ, Guccione AA, Kazis LE (1992). AIMS2. The content and properties of a revised and expanded Arthritis Impact Measurement Scales Health Status Questionnaire. Arthritis Rheum.

[B27] Guillemin F, Coste J, Pouchot J, Ghezail M, Bregeon C, Sany J (1997). The AIMS2-SF: a short form of the Arthritis Impact Measurement Scales 2. French Quality of Life in Rheumatology Group. Arthritis Rheum.

[B28] Haavardsholm EA, Kvien TK, Uhlig T, Smedstad LM, Guillemin F (2000). A comparison of agreement and sensitivity to change between AIMS2 and a short form of AIMS2 (AIMS2-SF) in more than 1,000 rheumatoid arthritis patients. J Rheumatol.

[B29] Rosemann T, Korner T, Wensing M, Schneider A, Szecsenyi J (2005). Evaluation and cultural adaptation of a German version of the AIMS2-SF questionnaire (German AIMS2-SF). Rheumatology (Oxford).

[B30] Taal E, Rasker JJ, Riemsma RP (2004). Sensitivity to change of AIMS2 and AIMS2-SF components in comparison to M-HAQ and VAS-pain. Ann Rheum Dis.

[B31] Moseley JB, O'Malley K, Petersen NJ, Menke TJ, Brody BA, Kuykendall DH, Hollingsworth JC, Ashton CM, Wray NP (2002). A controlled trial of arthroscopic surgery for osteoarthritis of the knee. N Engl J Med.

[B32] Keefe FJ, Lefebvre JC, Egert JR, Affleck G, Sullivan MJ, Caldwell DS (2000). The relationship of gender to pain, pain behavior, and disability in osteoarthritis patients: the role of catastrophizing. Pain.

[B33] Sharma L, Cahue S, Song J, Hayes K, Pai YC, Dunlop D (2003). Physical functioning over three years in knee osteoarthritis: role of psychosocial, local mechanical, and neuromuscular factors. Arthritis Rheum.

[B34] Dieppe P, Brandt KD (2003). What is important in treating osteoarthritis? Whom should we treat and how should we treat them?. Rheum Dis Clin North Am.

[B35] Craig CL, Marshall AL, Sjostrom M, Bauman AE, Booth ML, Ainsworth BE, Pratt M, Ekelund U, Yngve A, Sallis JF, Oja P (2003). International physical activity questionnaire: 12-country reliability and validity. Med Sci Sports Exerc.

[B36] Rutten A, Abu-Omar K (2004). Prevalence of physical activity in the European Union. Soz Praventivmed.

[B37] Pate RR, Pratt M, Blair SN, Haskell WL, Macera CA, Bouchard C, Buchner D, Ettinger W, Heath GW, King AC, . (1995). Physical activity and public health. A recommendation from the Centers for Disease Control and Prevention and the American College of Sports Medicine. JAMA.

[B38] KELLGREN JH, LAWRENCE JS (1957). Radiological assessment of osteo-arthrosis. Ann Rheum Dis.

[B39] McCullagh P NJA (1989). Generalized Linear Model.

[B40] LC H (1992). Regression with graphics: A second course in applied statistics.

[B41] Abdel-Nasser AM, Abd El-Azim S, Taal E, El Badawy SA, Rasker JJ, Valkenburg HA (1998). Depression and depressive symptoms in rheumatoid arthritis patients: an analysis of their occurrence and determinants. Br J Rheumatol.

[B42] Doeglas DM, Suurmeijer TP, van den Heuvel WJ, Krol B, van Rijswijk MH, van Leeuwen MA, Sanderman R (2004). Functional ability, social support, and depression in rheumatoid arthritis. Qual Life Res.

[B43] Graney MJ (2000). The reciprocal relationship between disability and depression. J Am Geriatr Soc.

[B44] Creamer P, Hochberg MC (1998). The relationship between psychosocial variables and pain reporting in osteoarthritis of the knee. Arthritis Care Res.

[B45] Penedo FJ, Dahn JR (2005). Exercise and well-being: a review of mental and physical health benefits associated with physical activity. Curr Opin Psychiatry.

[B46] Creamer P, Lethbridge-Cejku M, Hochberg MC (1999). Determinants of pain severity in knee osteoarthritis: effect of demographic and psychosocial variables using 3 pain measures. J Rheumatol.

[B47] Tsai PF, Tak S, Moore C, Palencia I (2003). Testing a theory of chronic pain. J Adv Nurs.

[B48] Ethgen O, Vanparijs P, Delhalle S, Rosant S, Bruyere O, Reginster JY (2004). Social support and health-related quality of life in hip and knee osteoarthritis. Qual Life Res.

[B49] Sherman AM (2003). Social relations and depressive symptoms in older adults with knee osteoarthritis. Soc Sci Med.

[B50] Rene J, Weinberger M, Mazzuca SA, Brandt KD, Katz BP (1992). Reduction of joint pain in patients with knee osteoarthritis who have received monthly telephone calls from lay personnel and whose medical treatment regimens have remained stable. Arthritis Rheum.

[B51] Keefe FJ, Blumenthal J, Baucom D, Affleck G, Waugh R, Caldwell DS, Beaupre P, Kashikar-Zuck S, Wright K, Egert J, Lefebvre J (2004). Effects of spouse-assisted coping skills training and exercise training in patients with osteoarthritic knee pain: a randomized controlled study. Pain.

[B52] Felson DT, Anderson JJ, Naimark A, Walker AM, Meenan RF (1988). Obesity and knee osteoarthritis. The Framingham Study. Ann Intern Med.

[B53] Felson DT, Zhang Y, Anthony JM, Naimark A, Anderson JJ (1992). Weight loss reduces the risk for symptomatic knee osteoarthritis in women. The Framingham Study. Ann Intern Med.

[B54] Szoeke C, Dennerstein L, Guthrie J, Clark M, Cicuttini F (2006). The relationship between prospectively assessed body weight and physical activity and prevalence of radiological knee osteoarthritis in postmenopausal women. J Rheumatol.

[B55] Klinger L, Spaulding SJ, Polatajko HJ, MacKinnon JR, Miller L (1999). Chronic pain in the elderly: occupational adaptation as a means of coping with osteoarthritis of the hip and/or knee. Clin J Pain.

[B56] Leigh JP, Fries JF (1994). Correlations between education and arthritis in the 1971-1975 NHANES I. Soc Sci Med.

[B57] Felson DT, Naimark A, Anderson J, Kazis L, Castelli W, Meenan RF (1987). The prevalence of knee osteoarthritis in the elderly. The Framingham Osteoarthritis Study. Arthritis Rheum.

[B58] Escalante A, del R (1999). How much disability in rheumatoid arthritis is explained by rheumatoid arthritis?. Arthritis Rheum.

